# Mpox, Caused by the MPXV of the Clade IIb Lineage, Goes Global

**DOI:** 10.3390/tropicalmed8020076

**Published:** 2023-01-20

**Authors:** Liping Gao, Qi Shi, Xiaoping Dong, Miao Wang, Zhiguo Liu, Zhenjun Li

**Affiliations:** 1 National Institute for Viral Disease Control and Prevention, Chinese Center for Disease Control and Prevention, 155 Changbai Road, Changping, Beijing 102206, China; 2 Ulanqab Center for Disease Control and Prevention, Jining 102206, China; 3 National Institute for Communicable Disease Control and Prevention, Chinese Center for Disease Control and Prevention, 155 Changbai Road, Changping, Beijing 102206, China; 4 Vocational and Technical College, College of Veterinary Medicine, Inner Mongolia Agricultural University, Baotou 014109, China

**Keywords:** Mpox, MPXV, temporal trend, exported cases, West Africa, clade IIb

## Abstract

Mpox is a great public health concern worldwide currently; thus, a global primary epidemiological analysis of mpox and a phylogenetic analysis of currently circulating MPXV strains based on open-source data is necessary. A total of 83,419 confirmed cases with 72 deaths were reported from 7 May to 23 December 2022, representing an ongoing increasing trend. Mpox was largely restricted to being endemic in children in West Africa (WA) before 2022, and it mainly spread from animals to humans. Our analysis highlights that mpox has not only spread across regions within Africa but has also led to most infection events outside Africa. Currently, mpox has been dominated by human-to-human spread in 110 countries, with the majority of cases distributed in the non-endemic regions of Europe and North America. These data indicate that the geographic range, transmission route, vulnerable populations, and clinical manifestations of mpox have changed, which suggests that the niche of mpox has the potential to change. Remarkably, approximately 38,025 suspected mpox cases were recorded in West and Central Africa during 1970–2022, which implied that the epidemiology of mpox in the two regions remained cryptic, suggesting that strengthening the accuracy of molecular diagnosis on this continent is a priority. Moreover, 617 mpox genomes have been obtained from 12 different hosts; these data imply that the high host diversity may contribute to its ongoing circulation and global outbreak. Furthermore, a phylogenetic analysis of 175 MPXV genome sequences from 38 countries (regions) showed that the current global mpox outbreak was caused by multiple sub-clades in the clade IIb lineage. These data suggest that MPXV strains from the clade IIb lineage may play a predominated role in the spread of mpox worldwide, implying that the current mpox outbreak has a single infection source. However, further investigations into the origin of the new global mpox outbreak are necessary. Therefore, our analysis highlights that adjusted timely interventive measures and surveillance programs, especially using cheap and quick strategies such as wastewater monitoring the DNA of MPXV in Africa (WA), are important for uncovering this disease’s transmission source and chain, which will help curb its further spread.

## 1. Introduction

Mpox, a rare viral zoonotic re-emerging disease caused by an orthopoxvirus, results in a smallpox-like disease in humans [1,2]. Human MPXV infections historically arise from animal-to-human transmission, and various animal species are susceptible to infection with MPXV, including a range of rodents and non-human primates [3]. Since the first human mpox case was recorded in 1970 in the Democratic Republic of the Congo (DRC), it has cross-border spread to other African countries, especially countries in West and Central Africa, and even to countries outside Africa in recent years [4,5,6]. It has similar clinical signs and symptoms as smallpox and a fatality rate of 11% in unvaccinated patients [7]. Mpox typically presents clinically with fever and rash, and may lead to a range of medical complications [8]. It has been reported that mpox cases have a substantial association with meteorological factors, such as temperature, dew/frost point, precipitation, relative humidity, and wind speed [9]. Vaccines based on the vaccinia virus have historically been used to prevent and eradicate smallpox, and the same vaccine (ACAM2000) is effective in controlling MPXV. It has been reported that the Modified Vaccinia Ankara-Bavarian Nordic is 85% effective in protecting against MPX [10,11]. Generally, the infection is self-limiting, with symptoms lasting from 2 to 4 weeks; however, it can be occasionally fatal (3–6 % fatality rates) [12]. Currently, two antiviral drugs, i.e., tecovirimat (TPOXX) and Brincidofovir, have been approved for treatment options for critically sick and immunocompromised infected individuals [13].

The MPXV is a large, enveloped, and brick-shaped double-stranded DNA virus that belongs to the *Orthopoxvirus* genus of the *Poxviridae* family [14]. Based on the genetic and geographic location first detected, there are two recognized phylogenetic branches, i.e., the central African (Congo Basin) clade and the West African clade [15]. According to the latest nomenclate, the former Congo Basin (Central African) clade is Clade one (I) and the former West African clade is Clade two (II) [16]. Additionally, it was agreed that Clade II consists of two subclades (IIa and IIb). There was an approximately 900 bp genomic length difference observed between strains from the clades lade I and clade II lineage [17]. In addition, clade I is considered to cause more severe symptoms and is more contagious. However, real-time PCR is currently best used in a major laboratory, thus limiting its use as a real-time diagnostic in rural, resource-poor areas, especially in Africa, making surveillance in mostly rural areas with poor infrastructure difficult [13]. Moreover, the number of cases detected outside Africa in the past few months alone has already surpassed the total number detected outside the continent since 1970 [18]. Therefore, an epidemiological analysis of Africa (WA) and global level of mpox is necessary to map the epidemic and understand how it spreads. In the present study, our purpose is to analyze the worldwide mpox epidemic profile and to summarize the dominant MPXV lineage for better understating the mpox outbreak and to provide valuable insight.

## 2. Methods

### 2.1. Data Sources

We retrieved epidemiologic data from the “WHO AFRO Weekly Bulletin on Outbreaks and Other Emergencies” (https://worldhealthorg.shinyapps.io/mpx_global/, accessed on 23 December 2022) [8], “Our World in Data”, and Gisaid.org (https://www. gisaid.org/, accessed on 23 December 2022), as well as from published literature. All of the data were processed and cross-checked by two trained qualified health workers. Microsoft Excel (Microsoft Office 2016, Microsoft Corporation, Redmond, WA, USA) was used for processing, analyzing, and plotting filtered data. Moreover, a Data wrapper (https://www.datawrapper.de/, accessed on 23 December 2022) was applied to create the figure for the geographic distribution of confirmed mpox cases.

### 2.2. Phylogenetic Analysis of 175 Genomes of MPXV

A total of 528 mpox genomes (as of 3 October 2022) from six continents were selected and used for phylogenetic analysis based on the online analysis tool (built with nextstrain/monkeypox (https://nextstrain.org/mpox/mpxv, accessed on 23 December 2022)). Subsequently, to better display the results of genome analysis, a total of 175 MPXV genomes were selected (Table S1) in GenBank from 38 countries (regions) based on the time order and clade types during the period 1961–2022 for the phylogenetic analysis. With monkeypox virus GCA_006458845.1 as a reference genome, genomic alignment between the sample genome and reference genome (GCA_006458845.1) was performed using the MUMmer [19] and LASTZ [20] tools. Whole-genome SNPs were found using the results of genomic alignment among samples by the MUMmer and LASTZ, as mentioned above. The phylogenetic tree was constructed using TreeBeST (Version 1.9.2) (https://github.com/Ensembl/treebest, accessed on 25 December 2022) with the approximately maximum-likelihood algorithm and 1000 replication bootstraps. Visualization of trees and editing was conducted using iTOL v6.6 (https://itol.embl.de/, accessed on 25 December 2022) [21]. Moreover, all software was used according to the default parameter settings.

## 3. Results

### 3.1. Persistent Geographic Expansion of Mpox Worldwide

**Figure 1:**
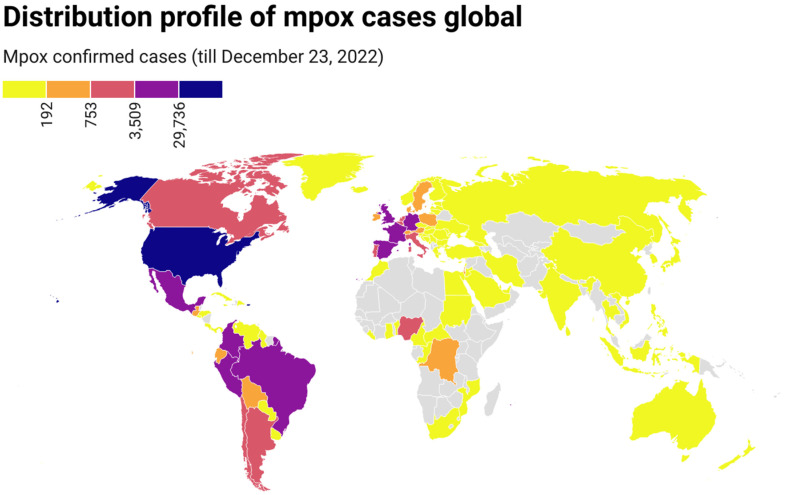
Geographic distribution of confirmed mpox cases from 7 May to 23 December 2022 (https://datawrapper.dwcdn.net/1NRBR/5/, accessed on 23 December 2022).

On 7 May 2022, the first indigenous mpox case was reported in the UK, which belonged to clade II [22]. By 23 December 2022, mpox had been detected in 110 countries from 6 continents, and 83,419 confirmed mpox cases with 72 deaths had been reported worldwide (Table S2). The number of cases increased continuously with time, although there were great fluctuations on some days. The mpox epidemic continues to expand globally. Confirmed mpox cases have been reported in many non-endemic countries from Europe, America, Asia, Oceania, and Africa (countries), where mpox was not usual or had not previously been reported (Figure 1). Of these cases, most were from North America (35,201, 42.20%), followed by Europe (25,684, 36.10%), South America (21,005, 25.18%), Africa (1199, 1.44%), Oceania (186, 0.22%), and Asia (144, 0.17%). Regarding dates, most cases (*n* = 1814) were reported on August 10, 2022, followed by September 21, 2022 (*n* = 1703), October 5, 2022 (*n* = 1663), and August 24, 2022 (*n* = 1658). Regarding countries, most cases were reported in the United States (*n* = 29,528), followed by Brazil (*n* = 10,375), Spain (*n* = 7496), France (*n* = 4,110), Colombia (*n* = 3971), the United Kingdom (*n* = 3730), and Germany (*n* = 3676) (Figure 1).

### 3.2. Age, Clinical Symptoms, and Transmission Profile in Available Data

In terms of age, 96.6% (71,598/74,102) of cases with available data were male, and the median age was 34 years [8]. In addition, more than 79.2% of male cases were in the age range of 18–44 years, of which the 30–39 age group accounted for 40% (29,673/73,994), and cases in this age group were the most affected in the present mpox outbreak. Of the 79,583 cases where age was available, approximately 1.0% (779/79,583) of cases was aged 0–17, and the majority of cases aged 0–17 was from the non-endemic region of the Americas (626/779, 80%) [8]. Among all the confirmed cases, 79.2% showed any rash (systemic, oral, genital, or unknown location), with 57.1% having fever, 49.8% showing systemic rash, 44.6% showing genital rash, and 31.45% have a headache. Transmission data were available for only 26.5% (21,121/79,787) of cases, and 69.2% (14,615 of 21,121) of transmission events were reported to be sexual encounters. Of all settings in which cases were likely exposed, 67.1% (3367 of 5019) of cases were party settings with sexual contacts in likely exposure categories. Generally, the severity extent of the disease has been low; only 0.086% (72/83,419) of cases led to hospitalizations and deaths [8].

### 3.3. Trends of Mpox Cases in West and Central Africa

**Figure 2:**
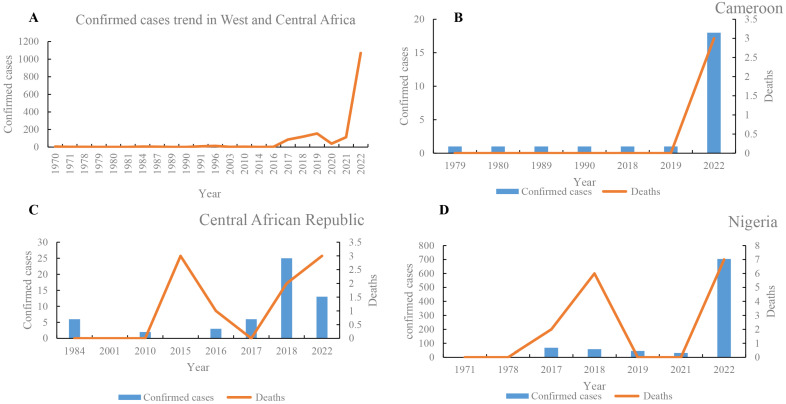
Comparative analysis of trends of mpox cases in West and Central Africa. Confirmed cases trends of mpox cases in West and Central Africa (**A**), and Cameroon (**B**), Central African Republic (**C**), and Nigeria (**D**), respectively.

In West and Central Africa, the number of mpox cases has increased continuously (Figure 2), and the epidemic geography has gradually expanded. The first case of mpox was reported in 1970 in Africa [23]. From 1970 to 23 December 2022, the number of mpox suspected, confirmed, and death cases in West and Central Africa were at least 38,025, 3849, and 576, respectively (Table S3). The number of mpox suspected, confirmed, and death cases in 2020 were 6661 (0.175%), 1069 (0.28%), and 20 (0.035%), respectively. These data demonstrate that there was a significantly increased trend in 2020. In addition, the epidemic geography of mpox has gradually expanded from nine countries in the 1970s to the most involved 13 countries, including two newly expanded countries (Gabon and Central African Republic) in 1980, one (Republic of the Congo) in the 2000s, and another one (Morocco) in 2022. The number of suspected cases was highest in the DRC (*n* = 35,678), followed by Nigeria (*n* = 1206) and the Central African Republic (*n* = 190) (Table S3). Likewise, the number of confirmed cases was highest in the DRC (*n* = 2203), followed by Nigeria (*n* = 908) and Ghana (*n* = 116). Moreover, of 576 reported deaths, 527 cases (91.50%) were reported in the DRC (of which 503 deaths were recorded during 2018–2022), followed by 15 deaths in Nigeria. In WA, the prevalence of mpox was highest in Nigeria, where five large-scale mpox outbreaks were recorded in 2017–2022 (Table S3). In Nigeria, 1206 suspected cases, 908 confirmed cases, and 15 deaths were reported from 1971 to 2022 (Figure 2). Moreover, intermittent cases were recorded in Sierra Leone in 1970, 2014, 2017, and 2021. Between 1 January and 23 December 2022, there were 1202 confirmed cases of mpox and 16 deaths due to mpox in Central and WA (Table S3). The confirmed cases in 2020 were mainly distributed in Nigeria (*n* = 704), the DRC (*n* = 206), and Ghana (*n* = 116). Moreover, more than 6000 suspected cases were reported in 2020, revealing that the true epidemic of mpox in West and Central Africa remains unclear, and needs to be further investigated.

### 3.4. The Role of West African Mpox in the Global Spread

To date, most mpox infection events outside Africa have been directly or indirectly caused by mpox from WA (Table 1). A total of 20 exported mpox infection events were recorded, of which 15 came from WA. Mpox in WA also has cross-region transmission within Africa. The earliest exported case within WA, a patient from Benin who had contracted the infection in Oyo State, Nigeria, was reported in 1978. Moreover, 10 mpox cases were exported from the DRC to South Sudan in 2005. Likewise, 10 mpox cases (8 suspected and 2 confirmed) were found, which were caused by refugee migration from the DRC to the Republic of the Congo in 2010. In 2003, the first exported mpox case from Ghana (WA) to the USA was reported, which was linked to contact with infected pet prairie dogs. At least 15 exported events were observed during 2018–2022, increasing annually. The countries reporting imported infection included Israel, the UK, Singapore, the USA, and the United Arab Emirates; of them, the highest number of exported infection events occurred in the UK (*n* = 9). During 2018–2022, the majority of cases were exported from Nigeria, including cases reported in Benin, Israel, the UK, Singapore, and the USA. Moreover, the first confirmed imported mpox case in the United Arab Emirates was reported on 24 May 2022, in a patient with a travel history to WA. Travel history to WA (Nigeria) is an important risk factor, and human-to-human transmission is the main infection route.

### 3.5. Geographical Distribution and Host Distribution of Mpox Genomes and Phylogenetic Analysis

As of 23 December 2022, 617 mpox genomes from six continents have been published (https://nextstrain.org/mpox/mpxv, accessed on 23 December 2022), among which the number per clade area was as follows: I (*n* = 48), IIa (*n* = 26), and IIb (*n* = 543). These data suggest that clade IIb lineages are a predominant circulating population. The numbers of published genomes per continent are ordered as follows: Europe (*n* = 330), North America (*n* = 111), Africa (*n* = 103), South America (*n* = 62), Asia (*n* = 9), and Oceania (*n* = 1). Mpox genomes from 41 countries have been published. The most genomes were published by the USA (*n* = 60) and United Kingdom (*n* = 60), followed by Portugal (*n* = 51), Canada (*n* = 50), Germany (*n* = 50), Colombia (*n* = 50), Spain (*n* = 47), Slovenia (*n* = 36), the DRC (*n* = 35), and Nigeria (*n* = 33). The remaining 31 countries published 1 to 23 different genomes. Furthermore, mpox sequences have been obtained from 12 different hosts (https://nextstrain.org/mpox/mpxv), including *Homo sapiens* (*n* = 570), *Pan troglodytes verus* (*n* = 12), *Cricetomys Gambians* (*n* = 1), *Crocidura littoralis* (*n* = 1), *Cynomys* (*n* = 1), *Funisciurus* (*n* = 1), *Funisciurus anerythrus* (*n* = 1), *Funisciurus bayonii* (*n* = 1), Gliridae (*n* = 1), *Malacomys longipes* (*n* = 1), and Platyrrhini (*n* = 1). These data imply that the high host diversity may contribute to its ongoing circulation and global outbreak.

**Figure 3:**
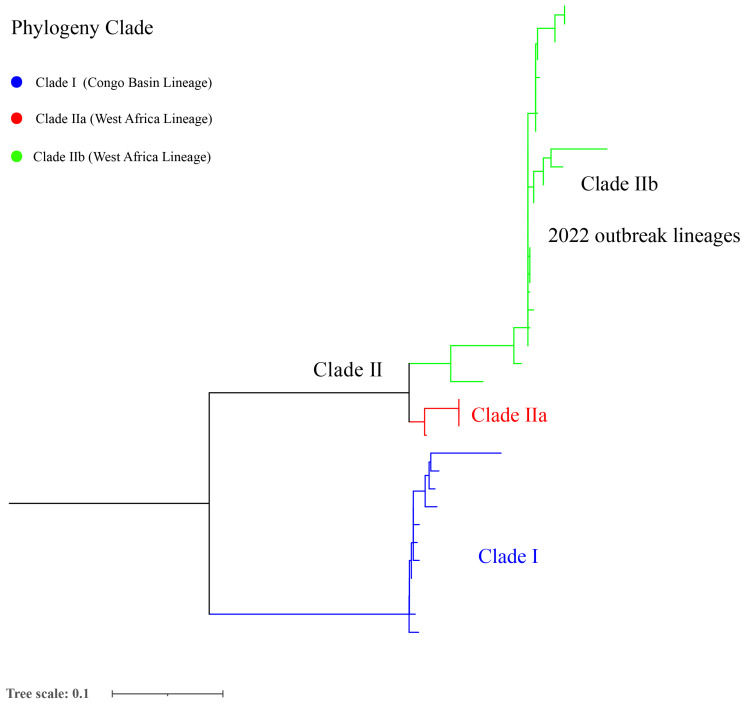
Phylogenetic analysis of 175 MPXV selected genomes from a global level.

According to the comparison with the reference genome, the total SNPs range of strains was 6 (AY753185.1) to 754 (NC_003310.1) among 175 selected mpox genomes (Table S4). Furthermore, the phylogenetic analysis showed that the 2022 mpox global outbreak was caused by strains from many new sub-clades belonging to clade IIb, which potentially reflects the continuous evolution characteristic of clade II (Figure 3 and Figure S1).

## 4. Discussion

In the present study, we conducted a comprehensive analysis of the geographic and temporal distribution changes in global mpox and SNP phylogenetic analysis of 175 MPXV genomes from 38 different countries (regions). Our analysis highlights that mpox has not only spread across regions within Africa, but has also led to most infection events outside Africa historically. MPXV was rarely seen outside the African continent. It has generally spread beyond the African continent due to the importation of animals and international travel [37]. Although mpox is known to be endemic in 11 African countries, the number of cases is grossly underestimated due to lack or poor laboratory surveillance/weak routine detection and other confounders including asymptomatic infection, sexual transmission, and the role of mass gatherings in sub-Saharan Africa and elsewhere [38]. Moreover, poverty and vulnerability, malnutrition, and poor healthcare behavior have been closely linked to the emergence and re-emergence of infectious diseases in low-income countries mainly in sub-Saharan Africa. Furthermore, regarding the causes of MPXV re-emergence, especially in endemic regions and developing countries, there are insufficient data about transmission routes and potential reservoir hosts, inadequate skills and experiences of health workers, expensive detection methods, and lack of public health intervention strategies [39]; these factors make the African continent a hotspot for vector-borne and zoonotic viral diseases that may spread globally [40]. There are other viruses to keep in mind, such as the recent re-emergence of Marburg virus disease in Ghana [41], the Langya virus [42], and the ongoing COVID-19 pandemic [43], which has resulted in serious health concerns globally. However, wastewater-based surveillance (WBS) of MPXV DNA has been extensively utilized to monitor MPXV and SARS-CoV-2 [44,45,46] and supports the possibility of using WBS as an early proxy for the detection of MPXV infections [3,47]. Therefore, strengthening the detection technology and funding support in Africa is recommended, which will be beneficial to clarify the reason for the current outbreak.

The present study highlighted that mpox’s geographic areas affected by the epidemic persistently expanded, and human-to-human transmission has become the primary mode of transmission during the current outbreak [48]. Multiple risk factors may co-drive the current mpox outbreak spread to many continents, and the changes in its niche [49], including the waning of population immunity to mpox, changes in ecosystems, the wide host range of the virus, human–wildlife interactions, undetected circulation in wildlife in pan-geographic areas, and better-adapted strains of the virus, might be contributing to the changing epidemiology of MPXV [50]. Moreover, climate changes and resource availability have been shown to play important role in changing viral dynamics and in the increased incidence of zoonoses [51]. In addition, apolipoprotein B mRNA-editing catalytic polypeptide-like 3 (APOBEC3) enzymes can be triggers for MPXV adaptive evolution toward enhanced transmissibility [52], and some gene loss events of the MPXV have been correlated with human-to-human transmission [53]. Differences in the MPXV ecology and host cell signaling responses in West and Central Africa [54,55], some different genes (N2R and N3R) [53], and the Golgi-associated retrograde protein (GARP) complex [56] were associated with the human-to-human transmission; however, further research is warranted. There have been multiple introductions from animal reservoirs to the human population, and human-to-human transmission has also occurred [57]. These factors suggest that the niche of mpox has the potential to change, and it is recommended that timely intervention measures and surveillance programs are implemented and adjusted. Moreover, travel plays a vital role in infectious disease outbreaks, especially traveling to susceptible areas which increases exposure risk, such as COVID-19 and HIV/AIDS [58,59], although the majority of cases recently identified had no obvious contact history with animals, and most cases were not part of an established chain of transmission that was travel-related or caused by contact with symptomatic people or animals. However, high background levels of OPXV antibodies have been found in MPXV-endemic areas, such as the DRC [60] and Uganda [61], suggesting that mpox transmission may have gone undetected [62]. Likewise, the inability to ascertain the source of the current outbreak or link cases to an endemic area indicates a similar pattern of undetected spread for a substantial period [63]. Continuous monitoring of international travelers that have symptoms of MPXV, especially with fever and rash by healthcare workers around the world [64]. Therefore, this uncharacterized transmission chain potentially plays a vital role in the spread of mpox, and tests upon travel are still a priority.

The present analysis showed that the age distribution, transmission route, and clinical manifestation of mpox have changed over time. Human monkeypox infections have been confirmed to be present in persons with a median age of 38 years, compared to what was observed in West and Central Africa, which might reflect the growth of the population with weakened or no immunity against MPXV infection [65]. The median age of patients was 37.0 years, and skin-to-skin contact during sex was the dominant mechanism of transmission of mpox in Spain [66]. Moreover, our analysis implies that the age of the patient population displayed an increasing trend toward adults, and the disease’s spread also shifted to unusual sexual transmission routes. Importantly, with atypical presentations in a majority of cases, low-level clinical suspicion of mpox will lead to more difficulty in early diagnosis, which could then trigger a larger outbreak. The high number of suspected mpox cases in endemic regions indicates that there was a potential gap in diagnosis and identification among cases in this continent. Globalization and frequent migration will be leading a further increase in the incidence of mpox cases [67]. Therefore, improved diagnosis and infection control measures are crucial to curb the spread of mpox.

Phylogenetic analysis showed that the current global mpox outbreak epidemic was primarily caused by MPXV of clade IIb. The lineage B.1 includes all MPXV genomes from the 2022 outbreak, and it has been estimated that the B.1 lineage emerged in Europe on 2 March 2022 [68]. A similar study showed that the clade IIb was primarily related to the 2022 outbreak of human mpox going globally, where clusters with clade IIa are closely related to the recently exported cases from Nigeria to the United Kingdom, Israel, and Singapore in 2018–2019, suggesting connections to a single case [69]. However, two clades (IIb and IIa) were segregated in a divergent phylogenetic branch, likely reflecting continuous accelerated evolution [52]. Similarly, from 2017 to 2022, MPXV’s mutation and spread suggest that this virus continues to evolve through point mutation in the genes according to the available sequence data [70]. Surprised, the current mpox sustained transmission event possibly preceded the outbreak in 2022 in Europe and has remained largely undetected; furthermore, the distinct genomic signatures suggest that this transmission chain may not be linked to the large outbreak of mpox which occurred in 2022 [71]. The outbreak cause and source of SARS-CoV-2/COVID-19 and mpox remains unclear, and the SARS, Influenza, MERS, and SARS-CoV-2/COVID-19 pandemics demonstrate that there is a perennial risk of pandemics [37]. Therefore, our suggestion that heightened awareness, increasing genome surveillance and the wider availability of molecular detection and diagnostics of mpox cases is necessary to elucidate the transmission chain and source of this disease.

## 5. Limitations

There were a few limitations in the present study. First, our data were collected from publicly published documents, so the results from our study may be affected by the original profile of documents. Second, due to the low detection and test capacity of mpox and the weak health system in some regions of Africa (WA) [72], the true epidemic situation in Africa may be more severe than presented here. Third, few genomic sequences were published in Africa, restricting our understanding of the epidemic of WA and global mpox.

## 6. Conclusions

In the present study, we conducted a comprehensive analysis of the geographic as well as temporal distribution changes of mpox. The mpox outbreak was likely caused by the waning of population immunity, as well as changes to and destruction of both the habitat and environment of the disease’s reservoir; these factors triggered changes in the ecology and microevolution of the virus itself, raising virus spillover events. Our analysis provides new insights into the global mpox epidemic. Currently, the origin of the new global MPXV outbreak remains unknown, and ongoing and increasing outbreaks provide evidence that the most likely scenario is that cross-continent, cryptic human transmission has been ongoing for longer than previously thought. Therefore, implementing early screening of suspected cases and genome surveillance is important for curbing further spread.

## Figures and Tables

**Table 1:** Exported mpox from West and Central Africa.

Export Pattern	ID	Exporting Country	Importing Country	Time	No.	Transmission Route (Potential)	Ref.
Exported in Africa	1	DRC	South Sudan	2005	10	Unknown	[24]
	2		Congo	2010	2	Refugee migration	[24]
	3	Nigeria	Benin	1978	1	Close contact with a confirmed case	[24]
	4			2022.6	1	Travel history	[25]
	5			2022.6	1	Travel history	[25]
Exported outside Africa	1	Ghana	USA	2003	47	Close contact with pet dogs	[26]
	2	Nigeria	Israel	2018.9.17	1	Travel history	[27]
	3		UK	2018.9.7	1	Travel history	[28]
	4			2018.9.11	1	Travel history	[28]
	5			2018.9.26	1	Close contact with a confirmed case	[28]
	6			2019.12	1	Travel history	[29]
	7			2021.5.25	1	Travel history	[30]
	8			2021.5.31	1	Travel history	[30]
	9			2021.6.15	1	Travel history	[30]
	10			2022.5.6	1	Travel history	[31]
	11		Singapore	2019.5.8	1	Travel history	[32]
	12		USA	2021.7.15	1	Travel history	[33]
	13			2021.11.16	1	Travel history	[34]
	14	WA	United Arab Emirates	2022.5.24	1	Travel history	[35]
	15	Africa	USA	2022.5.26	1	Travel history	[36]

## Data Availability

The data presented in this study are available on request from the corresponding author.

## Supplementary Materials

The following supporting information can be downloaded at: https://www.mdpi.com/article/10.3390/tropicalmed8020076/s1, Figure S1: Phylogenetic analysis of 528 mpox genomes globally. Table S1: 175 monkeypox virus genome from GenBank used for phylogenetic analysis in the present study; Table S2: Geographic distribution of confirmed mpox cases from 1st May to 23rd December 2022; Table S3: Epidemic situation of mpox cases in West and Central Africa; Table S4: The SNPs profile of 175 mpox genome selected in the present study.
