# Supplementary figures and images for: Mpox, Caused by the MPXV of the Clade IIb Lineage, Goes Global
Source: Trop Med Infect Dis. 2023 Jan 20;8(2):76. doi: 10.3390/tropicalmed8020076 (PMC9966881; doi:10.3390/tropicalmed8020076)

Tree scale: 0.001

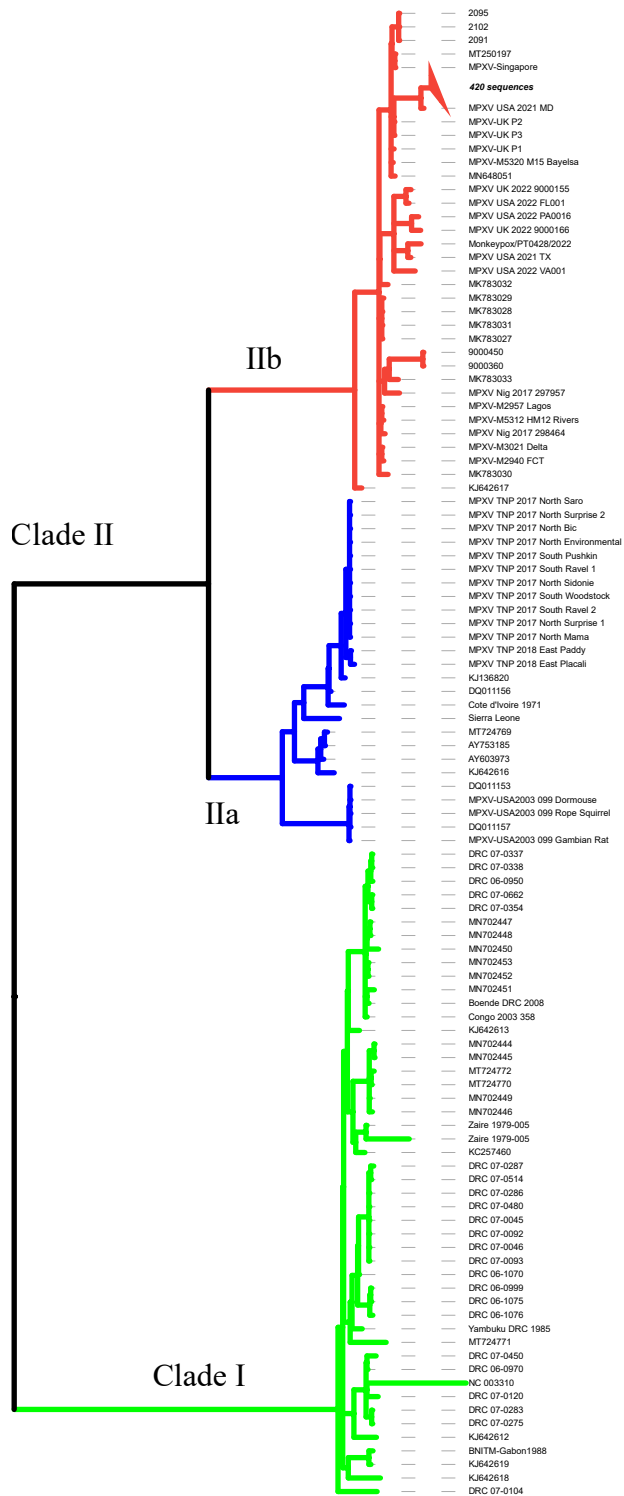

Supplement: Supplementary file 1 [file tropicalmed-08-00076-s001.zip › Figure S1.pdf]
